# Intravenous immunoglobulin for severe sepsis and septic shock: clinical effectiveness, cost-effectiveness and value of a further randomised controlled trial

**DOI:** 10.1186/s13054-014-0649-z

**Published:** 2014-12-01

**Authors:** Marta O Soares, Nicky J Welton, David A Harrison, Piia Peura, Manu Shankar-Hari, Sheila E Harvey, Jason Madan, Anthony E Ades, Kathryn M Rowan, Stephen J Palmer

**Affiliations:** Centre for Health Economics, University of York, Heslington, York, YO10 5DD UK; School of Social and Community Medicine, 39 Whatley Road, University of Bristol, Bristol, BS8 2PS UK; Intensive Care National Audit & Research Centre, 24 High Holborn, London, WC1V 6AZ UK; Guy’s and St Thomas’ Hospital NHS Foundation Trust, Westminster Bridge Road, London, SE1 7EH UK; University of Warwick, Coventry, CV4 7AL UK

## Abstract

**Introduction:**

Prior to investing in a large, multicentre randomised controlled trial (RCT), the National Institute for Health Research in the UK called for an evaluation of the feasibility and value for money of undertaking a trial on intravenous immunoglobulin (IVIG) as an adjuvant therapy for severe sepsis/septic shock.

**Methods:**

In response to this call, this study assessed the clinical and cost-effectiveness of IVIG (using a decision model), and evaluated the value of conducting an RCT (using expected value of information (EVI) analysis). The evidence informing such assessments was obtained through a series of systematic reviews and meta-analyses. Further primary data analyses were also undertaken using the Intensive Care National Audit & Research Centre Case Mix Programme Database, and a Scottish Intensive Care Society research study.

**Results:**

We found a large degree of statistical heterogeneity in the clinical evidence on treatment effect, and the source of such heterogeneity was unclear. The incremental cost-effectiveness ratio of IVIG is within the borderline region of estimates considered to represent value for money, but results appear highly sensitive to the choice of model used for clinical effectiveness. This was also the case with EVI estimates, with maximum payoffs from conducting a further clinical trial between £137 and £1,011 million.

**Conclusions:**

Our analyses suggest that there is a need for a further RCT. Results on the value of conducting such research, however, were sensitive to the clinical effectiveness model used, reflecting the high level of heterogeneity in the evidence base.

## Introduction

Sepsis is a clinical syndrome defined by the presence of both infection and a systemic inflammatory response; sepsis is defined as severe when associated with, or complicated by, organ dysfunction [1]. Severe sepsis may induce septic shock, defined as hypotension persisting despite adequate fluid resuscitation [2]. There is evidence indicating an increasing incidence of severe sepsis treated in critical care in England, Wales and Northern Ireland, rising from 50 to 70 cases per 100,000 population per year between 1995 and 2005 - these cases being associated with approximately 31,000 episodes of severe sepsis and 15,000 in-hospital deaths per year [3]. Being a serious, life-threatening condition, severe sepsis is expected to be associated with substantial healthcare costs and a significant impact on quality of life.

Intravenous immunoglobulin (IVIG) is a scarce blood product derived from human donor blood; it is currently subject to a Demand Management Programme by the United Kingdom (UK) Department of Health [4]. This product has been proposed as an adjuvant therapy for severe sepsis/septic shock since the 1980s. However, the mechanisms of action of IVIG are complex and are not yet fully understood. Despite this, a number of (predominantly small) randomised controlled trials (RCTs) have been conducted, and numerous systematic reviews and meta-analyses have been undertaken to synthesise their findings [5-8]. As a result of the heterogeneity across studies and the inconsistencies in their results, the majority conclude that there is currently insufficient evidence to recommend IVIG as an adjuvant therapy and that more evidence, in the form of a large, well-conducted RCT, is required.

Prior to investing in a large, multicentre RCT, the Health Technology Assessment (HTA) Programme of the National Institute for Health Research in the UK called for an evaluation of the feasibility and value for money of undertaking such a trial (that is, whether or not the costs of undertaking the trial are outweighed by the potential benefit of the resulting information). The aim of this manuscript is to report our findings in response to this call by assessing the clinical and cost-effectiveness of IVIG in severe sepsis/septic shock in adults, and evaluating the value of conducting a large, multicentre RCT using an expected value of information (EVI) analysis. EVI offers a methodological framework that explicitly considers the uncertainty surrounding the decision by a healthcare system to adopt a health technology and values the additional information, which may be generated by further research, in a way that is consistent with the objectives and resource constraints of heathcare provision [9].

A full technical report of this research is published elsewhere [10]. Here, we provide a summary of the evaluation of clinical effectiveness, cost-effectiveness, and value of information of IVIG in critically ill adults with severe sepsis/septic shock, undertaken to inform the UK policy context. We discuss the implications arising from this policy-driven evidence review.

## Methods

We conducted a series of formal systematic reviews and undertook additional primary data analysis to develop and populate a decision analytic model. Details of each review and data sources are presented in the full technical report [10]. The decision model evaluated the cost-effectiveness of IVIG as an adjunctive treatment to standard care for the management of adults with severe sepsis (we have used the term severe sepsis in this manuscript to include septic shock). The base-case population in the model reflected the baseline characteristics of the severe sepsis population in the Intensive Care National Audit & Research Centre (ICNARC) Case Mix Programme Database (CMPD), a high-quality clinical database of admissions to adult general (mixed medical/surgical) critical care units in the UK, considered to be more representative of current UK National Health Service (NHS) practice than the populations recruited into RCTs. We used data for the years 2007 to 2009, corresponding to a sample of 26,249 patients.

The decision model evaluated costs from the perspective of the NHS and Personal Social Services (PSS), and expressed these in British pounds sterling at a 2009 price base. Outcomes were expressed in quality-adjusted life years (QALYs). Both costs and outcomes were discounted using a 3.5% annual discount rate, in line with current guidelines [11]. The model was probabilistic, that is, uncertainty over the input parameters was propagated through the model in such a way that the results of the analysis could be presented with their associated uncertainty [12]. The expected costs and QALYs for IVIG and standard care (SC) were estimated and compared using incremental cost-effectiveness ratios (ICERs) that represent the incremental cost per additional QALY. The ICER was compared against thresholds used to establish value for money in the NHS (currently in the region of £20,000 to £30,000 per QALY) [11].

The probabilistic analysis also provided a formal approach to quantifying the consequences associated with the uncertainty surrounding the model results, which were then used to inform the EVI analyses. The maximum amount the NHS should be willing to invest to reduce uncertainty in the decision can be informed by the expected value of perfect information (EVPI) [13,14]. The EVPI evaluates the expected cost of current uncertainty by accounting both for the probability that a decision based on existing evidence is wrong and for the magnitude of the consequences of making the wrong decisions. EVPI can be expressed at a population level based on the size of the population (yearly incidence = 33,160) and the number of years research is assumed useful (10 years). EVPI can also be estimated for individual parameters (or for groups of parameters) contained in the model, termed partial EVPI or EVPPI. Five groups of uncertain parameters were considered: i) baseline mortality during the initial acute hospitalisation with SC; ii) clinical effectiveness of IVIG; iii) long-term mortality estimates for survivors of severe sepsis; iv) long-term costs for survivors of severe sepsis; v) quality of life for survivors of severe sepsis. The groups of parameters also reflect potentially different research designs. For example, while an RCT would ideally be required to further inform the clinical effectiveness of IVIG, evidence on the other parameters could be generated using record linkage of existing databases.

The EVPI estimates set an upper limit on the returns to further research. However, to fully inform the research decision, the most efficient research design also needs to be established, for example, the type of study, the optimal sample size, the appropriate duration of follow-up and appropriate end points. The same framework of analyses can be extended to establish the expected value of sample information (EVSI) for a particular research design. To obtain the societal payoff to the proposed research, the population EVSI needs to be compared with the costs of sampling: the difference between the EVSI and the costs of sampling gives the expected net benefit of sampling (ENBS). The ENBS provides a necessary and sufficient condition for deciding to conduct more research that is, if the ENBS is greater than zero for any sample size then the benefits of gathering the sample information exceed the costs, and further research is potentially justified. The ENBS also provides a framework for the efficient design of the clinical trial, where the optimal sample size, n^*^, for the proposed trial is where the ENBS reaches its maximum. This optimal sample size thus indicates how many patients should be enrolled for the trial to provide the highest payoff.

All stages of the work were informed through discussions with an expert clinical advisory group who provided feedback on specific aspects of the analyses including the model structure, inputs and assumptions.

### Decision model structure

**Figure 1:**
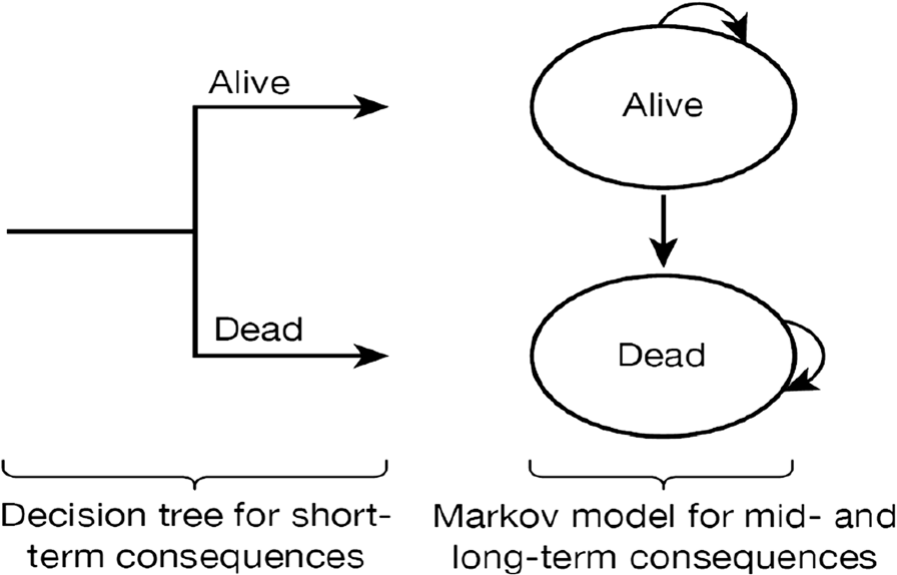
**Model schematic.**

A simplified schematic of the structure of the decision model is presented in Figure 1. The model evaluated the lifetime prognosis of severe sepsis in order to capture the longer-term costs and consequences associated with the natural history in the absence of IVIG. The model structure considered two related elements reflecting short- and long-term consequences:

i. short-term: the short-term consequences of the initial severe sepsis episode reflected the initial acute hospitalisation period (acute hospital and critical care) and related to the probability of surviving the initial acute hospitalisation. Baseline data from the CMPD were used to estimate the risk of acute hospital mortality associated with SC and the results of a systematic review of the clinical effectiveness of IVIG were applied to estimate the risk of acute hospital mortality associated with IVIG.
ii. longer-term: conditional on having survived the initial acute hospitalisation, a Markov structure was used to characterise the long-term prognosis over the remainder of a patient’s lifetime. Such a model represents disease progression by defining important mutually exclusive events/health states and characterising how patients may move between states over regular time intervals. Here, we used a simple structure to represent the possibility of patients being alive or dead (a survival model). Annual cycles were employed to reflect the annual probability of death for each year after the initial severe sepsis episode. Duration of stay alive or transition to death can be associated with a quality of life score and with costs incurred, allowing for long-term QALYs and costs to be evaluated. If IVIG reduces the risk of mortality during the initial acute hospitalisation period, then the use of this longer-term model will allow differences in long-term costs and QALYs to be translated.

The model was developed in the statistical programming package R [15].

### Clinical effectiveness of IVIG

Evidence on the effectiveness of IVIG was sought and used to inform the decision model and further analyses. A systematic review of the literature was conducted to inform the effect of IVIG in severe sepsis on all-cause mortality during the initial acute hospitalisation. Full details of the methods of the systematic review are reported in the full technical report [10]. In summary, RCTs within a critical care setting that compared any standard polyclonal IVIG or immunoglobulin M-enriched polyclonal IVIG (IVIGAM) with either no intervention, placebo (usually albumin) or another standard polyclonal IVIG or IVIGAM preparation were considered eligible for inclusion. Studies were included if the majority of patients were aged ≥18 years and clinical judgement deemed the population studied to have severe sepsis.

The primary outcome measure extracted was all-cause mortality, summarised on the odds ratio (OR) scale. Fixed- and random-effects meta-analyses were fitted using inverse variance weights. Heterogeneity was assessed using I^2^, and Cochrans Q test. Stata, version 11.0 (StataCorp LP, College Station, TX, USA), was used.

Heterogeneity was further explored comparing model fit from meta-regressions, estimated through Bayesian [16] Markov chain Monte Carlo simulation [17] using WinBUGS version 1.4.3 (MRC Biostatistics Unit, Cambridge, UK) [18,19]. More detail on the use of Bayesian methods in meta-analysis and evidence synthesis can be found in Sutton and Abrams [20], and in Spiegelhalter *et al.* [21]. Results are presented using means and 95% credible intervals, 95%CrI (the Bayesian equivalent to confidence intervals) [20]. The posterior mean residual deviance (Dres) was used to measure model fit and the deviance information criterion (DIC), a composite measure of model fit and model complexity, was used to choose between competing models [22]. For the random-effects models, the posterior mean of the between-study standard deviation (SD) parameter (τ) was used to investigate the impact of the inclusion of the covariates on explaining (reducing) heterogeneity. Meta-regressions aimed to: i) identify key covariates responsible for heterogeneity; ii) consider more complex treatment models that compared different types and preparations of IVIG; and iii) adjust for potential confounding by considering combinations of covariates. The covariates evaluated related to: characteristics of treatments such as type and dose of IVIG; features of study design related to, or a proxy for, study quality (for example Jadad score - a composite measure ranging from 0 to 5 where 5 indicates best quality and sample size); setting; acute severity of disease; and follow-up period. Key covariates that explained some of the heterogeneity using model fit statistics (Dres, DIC and τ) were identified. In addition, combinations of key potential covariates were explored to identify which of the covariates best explained the heterogeneity, after having adjusted for other covariates. Other treatment models were also explored, in which the type of IVIG preparation and type of control were not grouped together. All treatment and covariate models were compared using the model fit statistics (Dres, DIC and τ). Results were reported for the best-fitting, competing models.

### Other inputs of the decision model

#### *Baseline event rates for standard care* (*initial acute hospitalisation*)

Data from the CMPD (n = 26,249) were used to inform the baseline risk of acute hospital mortality applied to SC during the initial hospitalisation. Mortality risk was estimated by conditioning on characteristics of patients and the severity of illness at presentation: age and gender; Acute Physiology and Chronic Health Evaluation II (APACHE II) score; ICNARC physiology score and number of dysfunctional organ systems. Logistic regressions (robust standard errors (SEs) adjusting for clustering on critical care unit) were used.

#### Longer-term survival

We undertook additional primary data analysis to inform the longer-term survival estimates for severe sepsis survivors based on a cohort of 345 subjects from the Scottish Intensive Care Society (SICS) prospective, observational, multicentre, epidemiological study of severe sepsis [23]. Only patients (n = 271) for whom organ system dysfunction was clearly reported were selected. Average follow-up for survival was 787 days (range 0 to 2,062) days.

Parametric survival analyses were undertaken to estimate longer-term mortality (goodness of fit was assessed using Akaike information criterion (AIC) statistics). Three separate models were fitted including additional covariates for: age; APACHE II score at admission; and organ system dysfunction. The covariates were included to adjust for potential imbalances between the baseline characteristics from the CMPD (used to estimate short-term mortality) and the SICS study cohort. Also, these covariates allowed for consideration of subgroup-specific estimates for longer-term survival.

#### Resource use, unit costs and health-related quality of life

The length of stay (LOS) during the index severe sepsis episode (from the CMPD) was used to cost the acute hospital stay. Unit costs were derived from national databases. A summary of the information used is presented in Table 1 and further details of the results of two separate literature reviews, on health-related quality of life (HRQoL) and resource use, are available in the full technical report [10].

**Table 1:** **Inputs of the decision model for the evaluation of IVIG for severe sepsis/septic shock: parameter values and uncertainty over parameter values**

***Parameter***	***Source***	***Base case***	***Notes on scenario analyses undertaken***	***Notes on subgroup analyses***
*Cohort characteristics*				
Mean age of a severe sepsis patient at admission to hospital	CMPD	63 years old	Same as base case	Assumed to vary in subgroups defined using age. Sourced from ICNARC database
Proportion of males in a severe sepsis population at admission to hospital	CMPD	0.53	Same as base case	Assumed to vary in subgroups defined using gender. Sourced from CMPD
*Short-term outcomes* (*ST*)				
Probability of dying in hospital when SC is used in the treatment of severe sepsis (baseline risk)	CMPD	40.6%, 95% CI (40%, 41.2%)	Same as base case	Assumed to vary per subgroup (all). Sourced from CMPD
Odds ratio, when IVIG is used to complement SC in the treatment of severe sepsis (based on Model M1)	Evidence synthesis (‘Clinical effectiveness of IVIG’)	0.75 , 95% CI (0.58, 0.96)	Alternative models tested; see Table 2	Same as base case
*Longer-term outcomes* (*LT*)				
Age specific probability of dying in yearly intervals, conditional on patients having survived up to the start of the year.	Cuthbertson database and general population life tables 2010	Figure 4. Varies with time.	(1) time horizon	Assumed to vary for subgroups defined using age and APACHE II score. Sourced from CMPD
			(2) time points at which patients reverted to survival of general population	
*Cost-related parameters*				
Costs of overall IVIG therapy	Non-stochastic, BNF	£5,539.05	Same as base case	Same as base case
Costs of SC, when only SC is used in the treatment of severe sepsis	Non-stochastic,	£0	Same as base case	Same as base case
LOS in ICU for patients remaining alive until discharge from hospital	CMPD	8.48 (SE = 0.086)	Same as base case	Assumed to vary for all subgroups. Sourced from CMPD
LOS in ICU for patients dying in hospital	CMPD	7.40 (SE = 0.108)	Same as base case	Assumed to vary for all subgroups. Sourced from CMPD
Costs associated to a day in ICU for a patient with severe sepsis	Non-stochastic, reference costs [24]	£1,393	Same as base case	Same as base case
Overall hospital LOS for patients remaining alive until discharge from hospital	CMPD	21.29 (SE = 0.292)	Same as base case	Assumed to vary for all subgroups. Sourced from CMPD
Overall hospital LOS for patients dying in hospital	CMPD	39.07 (SE = 0.325)	Same as base case	Assumed to vary for all subgroups. Sourced from CMPD
Costs associated to a day in wards other than ICU for a patient with a severe sepsis episode	Non-stochastic, reference costs [24]	£196	Same as base case	Same as base case
Costs incurred between year *t-1* and year *t* after hospital discharge	Manns [25]	*t = 1*: £13,654 and *t >1*: £4,466.5/year	(1) ± 50% of Manns’ estimates	Same as base case
			(2) average annual per capital NHS cost for the general population	
*Utilities*				
In-hospital HRQoL weight associated to severe sepsis patients	Drabinsky [26]	0.53	Same as base case	Same as base case
HRQoL weight associated to severe sepsis patients at year *t*	Cuthbertson database, Drabinsky [26]	*t = 1*: 0.62 and *t >1*: 0.6833	Same as base case	Same as base case

See NIHR HTA full technical report [10] for a comprehensive reporting of parameter values used to inform the decision model, including those used in subgroup analyses. IVIG, intravenous immunoglobulin; CMPD, Case Mix Programme Database; ICNARC, Intensive Care National Audit & Research Centre; CI, confidence interval; BNF, British National Formulary; SC, standard care; APACHE II, Acute Physiology and Chronic Health Evaluation II; LOS, length of stay; HRQoL, health-related quality of life.

## Results

Table 1 provides a summary of the model input parameters used (both from the systematic reviews and from the primary data analysis) to inform the cost-effectiveness of IVIG.

### Clinical effectiveness of IVIG

**Figure 2:**
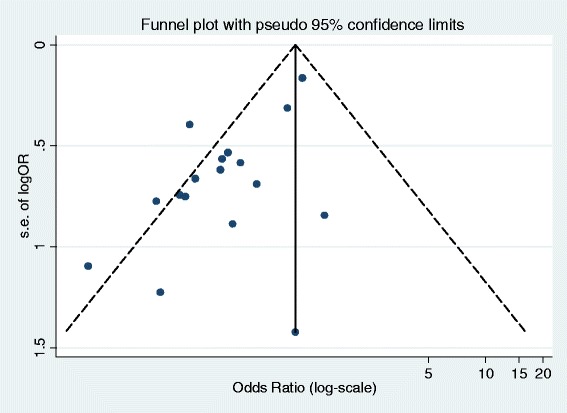
**Evidence on the clinical effectiveness of IVIG for severe sepsis (and septic shock): publication bias - funnel plot (with pseudo 95% confidence limits) for mortality of IVIG and IVGAM versus control.** IVIG, intravenous immunoglobulin; IVIGAM, immunoglobulin M-enriched polyclonal IVIG.

**Figure 3:**
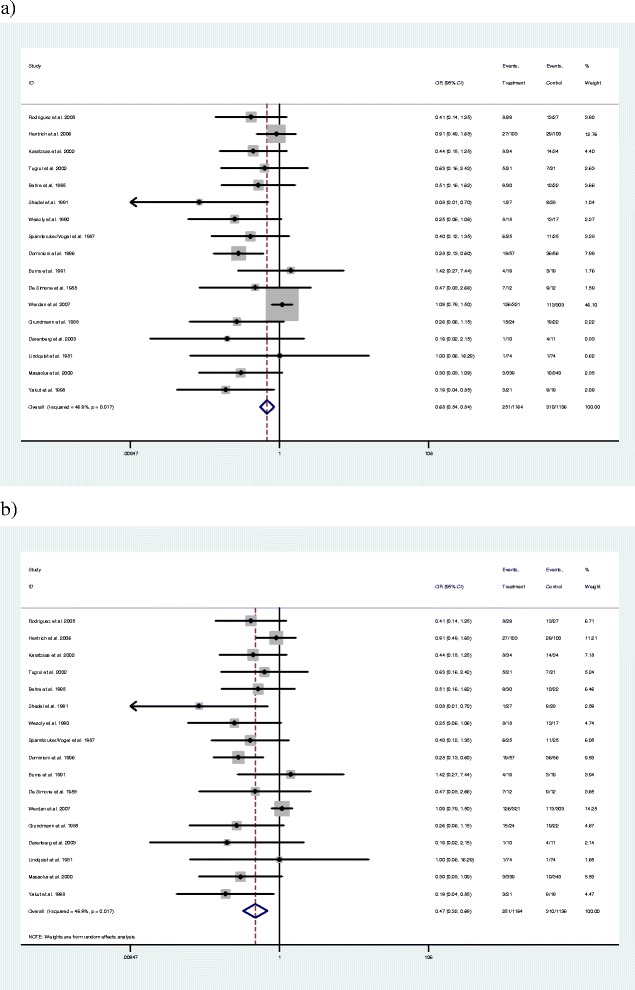
**Evidence on the clinical effectiveness of IVIG for severe sepsis (and septic shock): Forest plots for (a) fixed-effects model using inverse variance weights, and (b) random-effects model using inverse variance weights.** Both evaluate IVIG and IVIGAM treatments versus control. IVIG, intravenous immunoglobulin; IVIGAM, immunoglobulin M-enriched polyclonal IVIG.

Seventeen studies were identified that met the inclusion criteria with a large degree of heterogeneity in treatment effect between studies [27-40]. Figure 2 presents a funnel plot of the SE of the effect size (log-OR) plotted against study effect size (OR on the log scale). The asymmetry suggests there may be publication bias with this evidence and further exploratory analyses were conducted. Unadjusted fixed- and random-effects meta-analyses were implemented, initially comparing two treatment groupings (IVIG or IVIGAM vs. albumin/no treatment) (Figure 3). Given the heterogeneity (*I*^2^ = 46.9%), a random-effects model fitted the data well while the fixed-effect model showed substantial lack of fit. The pooled OR from the random-effects model was 0.47 (95% CI 0.32 to 0.69), showing a stronger effect than the fixed-effect model (pooled OR = 0.68; 95% CI 0.54 to 0.84).

From the meta-regression analyses, key covariates appearing to explain the existing heterogeneity between studies were: dosing regimen (duration of treatment (days), daily dose (g kg^−1^ day^−1^), volume (ml kg^−1^ day^−1^)); and study quality (that is use of albumin as control - as a proxy for proper blinding to treatment, Jadad score, publication date, and a measure of sample size; 1/ N). These two key covariates explained the majority of the heterogeneity in treatment effect across the studies. Further detail on these analyses is presented in the full technical report [10].

Across all the models considered [10], the best-fitting model assessed three treatments: IVIG/IVIGAM vs. albumin vs. no treatment and included duration of IVIG therapy as a treatment effect-modifying covariate. Results are reported for the most commonly used duration of therapy reported: three days (model M1 in Table 2). The OR for IVIG/IVIGAM vs. albumin was estimated at 0.75 (with a 95% CrI of 0.58 to 0.96) indicating a reduction in the odds of all-cause mortality for patients with severe sepsis compared with albumin.

**Table 2:** **Evidence on the clinical effectiveness of IVIG for severe sepsis/septic shock: estimates from the best-fitting models for the synthesis of evidence**

**Model for the synthesis of clinical effectiveness evidence**	**Odds ratio (95% CrI)**
**M1**: Fixed-effect model considering three treatments: IVIG/IVIGAM vs. albumin vs. no treatment, with covariate on duration of IVIG therapy. Relative effectiveness estimate reported for IVIG/IVIGAM vs. albumin for a duration of therapy of three days.	0.75 (0.58, 0.96)
**M2**: Random-effects model considering three treatments: IVIG/IVIGAM vs. albumin vs. no treatment, no covariates. Relative effectiveness estimate reported for IVIG/IVIGAM vs. albumin.	0.68 (0.16, 1.83)
**M3**: Random-effects model considering two treatments: IVIG/IVIGAM vs. albumin or no treatment, with covariate on Jadad score. Relative effectiveness estimate reported assuming a Jadad score of 5.	0.83 (0.18, 2.13)
**M4a**: Random-effects model considering two treatments: IVIG/IVIGAM vs. albumin/no treatment, with covariate representing $1/\sqrt{N}$. Relative effectiveness estimate reported assuming a sample size of 339 patients^*^	0.92 (0.23, 2.10)
**M4b**: Random-effects model considering two treatments: IVIG or IVIGAM vs. albumin or no treatment, with covariates representing $1/\sqrt{N}$ *.* Relative effectiveness estimate assumes an infinitely large sample size^*^	1.27 (0.25, 3.17)

^*^With model M4 two cases were considered: for M4a, the sample size N was set equal to the maximum arm size in the studies in our review - avoiding extrapolation beyond the dataset; for M4b, sample size was set to infinity; this demonstrates the effect on model estimates of the absence of bias associated with study quality, here proxied by finite (and small) sample sizes. CrI, credible interval, the Bayesian equivalent to confidence intervals; IVIG, intravenous immunoglobulin; IVIGAM, immunoglobulin M-enriched polyclonal IVIG.

Discussions with the expert clinical advisory group highlighted that there was no clear clinical rationale why duration of treatment would affect treatment effectiveness. For this reason, random-effects models with solely study quality covariates were also considered. The heterogeneity that can be explained with the dosing regimen covariates was left unexplained in these models, reflecting a belief that these covariates were a proxy for other, unmeasured, differences between the included studies. The results of a range of alternative models are presented in Table 2 (Models M2 to M4b).

When the heterogeneity explained by duration of IVIG therapy in M1 was treated as unexplained (that is using a random-effects model, models M2 to M4b), the majority of results were fairly comparable and still showed a reduction in the odds of all-cause mortality in patients with severe sepsis treated with IVIG but the 95% CrI widened suggesting a larger degree of uncertainty. The only exception was model M4b, which reported an increase in the odds of all-cause mortality, albeit with very wide credible intervals. However, some caution should be applied to this result since it involves extrapolation beyond the available data. In the absence of a single best-fitting model that made clinical sense, sensitivity analyses were subsequently used in the cost-effectiveness modelling.

### Other input parameters to the decision model

**Figure 4:**
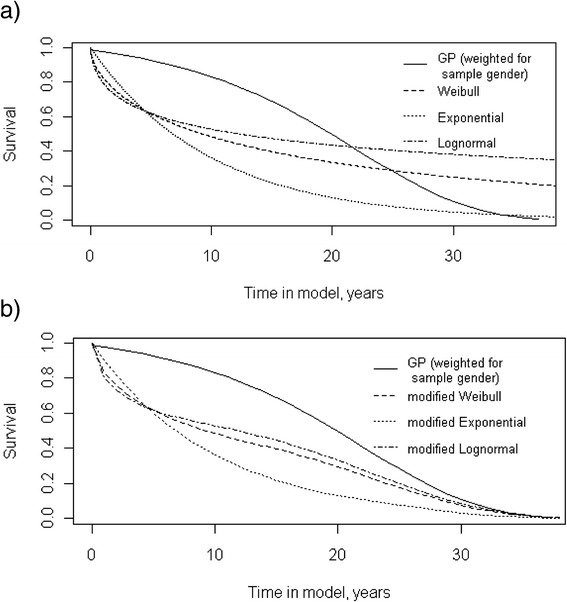
**Longer-term survival of patients after an acute episode of severe sepsis (or septic shock): comparison of parametric survival functions (a) and their modified versions (b) to general population.** The curves in Figure 3a investigate the plausibility of the different parametric (Weibull, Exponential and Lognormal) predictions beyond the five years of observed data by comparing these to age-adjusted estimates from the general population. These show that the longer-term mortality parametric estimates for severe sepsis patients become lower than that for the general population. This was considered implausible and, consequently, we modified these distributions by further assuming that the probability of mortality would be the maximum of the predicted parametric distributions and the observed yearly probability of mortality for the general population (age- and sex-adjusted). The ‘modified’ parametric survival functions are reported in Figure 4b.

Model input parameters and uncertainty around their estimates are described in Table 1 (and, in greater detail, in the full technical report [10]). Briefly, the probability of dying during the initial hospitalisation was estimated from the CMPD to be 40.6% (95% CI 40.0%, 41.2%). With respect to the evidence on longer-term mortality, we initially investigated the plausibility of the different parametric predictions beyond the five years of observed data by comparing these to age-adjusted estimates from the general population (Figure 4a). It was considered implausible that the longer-term mortality estimates for severe sepsis patients would become lower than that for the general population. Consequently, in the model, we further assumed that the probability of mortality would be the maximum of the predicted parametric distributions and the observed yearly probability of mortality for the general population (age- and sex-adjusted). The ‘modified’ parametric survival functions are reported in Figure 4b. The distribution with the best statistical goodness of fit was the Weibull function, and this was further used in the decision model [10].

With respect to resource use, we obtained estimates from the literature of total costs per annum and used these to describe costs after discharge from the index acute hospitalization [25]. The evidence used distinguishes the first year from subsequent years. With respect to HRQoL weights, we found evidence in the literature that the quality of life of survivors could be represented by a value of 0.69; also based on the available literature we assigned additional decrements to the within acute hospital period (0.09) and to the first month after acute hospitalisation (0.06).

### Cost-effectiveness of IVIG

Table 3 reports the cost-effectiveness results using the best-fitting clinical effectiveness model for all-cause mortality and for each of the alternative models from the clinical effectiveness review considered within the sensitivity analyses. The results for the best-fitting model show that the ICER of IVIG is £20,850 per QALY, which is within the borderline region of estimates considered to represent value for money in the NHS. At a threshold of £20,000 per QALY, the probability that IVIG is cost-effective is 0.505. As the threshold cost per QALY increased, the probability that IVIG is cost-effective increased (that is increasing to 0.789 at a threshold of £30,000).

**Table 3:** **Cost-effectiveness of IVIG for severe sepsis/septic shock using the best-fitting and alternative synthesis models of effectiveness evidence (see Table** 2 **for detailed specification of the models)**

(Best-fitting model, **M1)** Fixed-effect model estimate (IVIG/IVIGAM compared with albumin) considering 3 days duration of IVIG therapy				Probability of being cost-effective for cost-effectiveness threshold	
*Treatment*	*Mean cost*	*Mean QALY*	*ICER*	*£20,000/QALY*	*£30,000/QALY*
IVIG	£54,901	4.35	£20,850	0.505	0.789
Standard care	£45,593	3.90		0.495	0.211
(Alternative model **M2)** Random-effects model estimate (IVIG/IVIGAM compared with albumin)					
*Treatment*	*Mean cost*	*Mean QALY*	*ICER*	*£20,000/QALY*	*£30,000/QALY*
IVIG	£57,200	4.62	£16,177	0.597	0.707
Standard care	£45,593	3.90		0.403	0,295
(Alternative model **M3)** Random-effects model (IVIG/IVIGAM compared with albumin/no treatments) considering Jadad score = 5					
*Treatment*	*Mean cost*	*Mean QALY*	*ICER*	*£20,000/QALY*	*£30,000/QALY*
IVIG	£55,238	4.39	£19,968	0.502	0.611
Standard care	£45,593	3.90		0.498	0.389
(Alternative model **M4a)** Random-effects model (IVIG/IVIGAM compared with albumin/no treatment) considering a sample size of 339					
*Treatment*	*Mean cost*	*Mean QALY*	*ICER*	*£20,000/QALY*	*£30,000/QALY*
IVIG	£53,518	4.18	£28,520	0.404	0.514
Standard care	£45,593	3.90		0.596	0.486
(Alternative model **M4b)** Random-effects model (IVIG/IVIGAM compared with albumin/no treatment) considering a sample size of infinity					
*Treatment*	*Mean cost*	*Mean QALY*	*ICER*	*£20,000/QALY*	*£30,000/QALY*
IVIG	£50,024	3.76	Dominated	0.275	0.348
Standard care	£45,593	3.90		0.725	0.652

IVIG, intravenous immunoglobulin; IVIGAM, immunoglobulin M-enriched polyclonal IVIG; QALY, quality-adjusted life years; ICER, incremental cost-effectiveness ratio.

For the alternative clinical effectiveness models, the ICER estimates vary between £16,177 per QALY to IVIG being dominated by SC alone (that is IVIG being both less effective and more costly). These results clearly demonstrate that any conclusions regarding the cost-effectiveness of IVIG appear highly sensitive to the choice of model used for clinical effectiveness. Furthermore, the level of decision uncertainty, expressed in terms of the probability that IVIG is cost-effective, remains high across all these scenarios.

### Value of further information

Table 4 presents a summary of the population EVPI estimates based on a cost-effectiveness threshold of £20,000 per QALY. The results demonstrate a considerable range in the population EVPI estimates depending on the model applied to estimate the relative clinical effectiveness of IVIG. As expected, the random-effects model gave higher EVPI estimates given the additional between-study heterogeneity that is included. For a time horizon of 10 years, population EVPI varied between approximately £393 million and £1.4 billion. These results clearly suggested that further primary research would appear to be potentially worthwhile given the high cost of current decision uncertainty across all scenarios.

**Table 4:** **Value of further research on IVIG for severe sepsis/septic shock: population EVPI estimates (WTP = £20,000), according to alternative synthesis models of effectiveness evidence (see Table** 2 **for detailed specification of the models)**

**Scenarios for alternative synthesis models of effectiveness evidence**	**EVPI per patient**	**Population EVPI (** ***Time horizon*** **= 10 years)**
**M1**	£1,377	£392,994,216
**M2**	£3,563	£1,017,023,732
**M3**	£4,791	£1,367,426,550
**M4a**	£3,146	£897,945,285
**M4b**	£2,113	£603,018,958

EVPI evaluates the expected cost of current uncertainty by accounting both for the probability that a decision based on existing evidence is wrong and for the magnitude of the consequences of making the wrong decisions. EVPI, expected value of perfect information.

**Figure 5:**
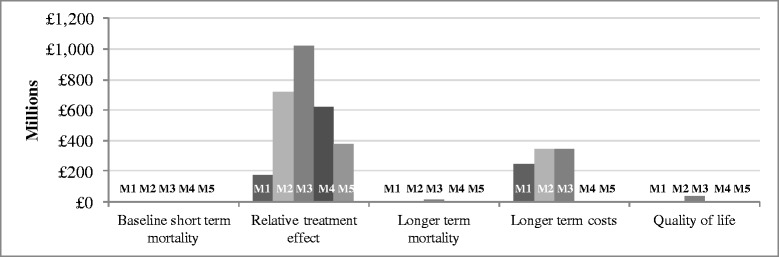
**Value of further research on IVIG for severe sepsis (and septic shock): population partial EVPI (EVPPI) for groups of uncertain parameters, according to model for the synthesis of effectiveness evidence (see Table** 2 **for detailed specification of the models).** IVIG, intravenous immunoglobulin; EVPI, expected value of perfect information; EVPPI, partial EVPI.

Figure 5 presents EVPPI estimates for the five groups of uncertain parameters for each of the clinical effectiveness models. The EVPPI associated with the relative treatment effect of IVIG consistently emerged as having significant influence on the overall decision uncertainty: the lowest estimate of EVPPI for the relative effect of IVIG was £173.7 million. The longer-term costs of severe sepsis also seem to be an important driver, with significant value in all except one of the scenarios. It should be appreciated that the costs of undertaking research on parameters such as quality of life would be significantly lower for these than for those required to undertake a large, multicentre RCT.

The population ENBS and the optimal sample size, n^*^, for a proposed trial are reported in Table 5 (for a threshold of £20,000). Calculations assumed the costs of the trial to be based on a fixed cost component of £2 million and a variable cost component of £2,000 per patient recruited (+ £5,500 for patients receiving IVIG). Across scenarios, the maximum payoff from conducting this research (the ENBS) varies between £137 and £1,011 million. The optimal sample size always exceeded 800 subjects for each arm.

**Table 5:** **Value of further research on IVIG for severe sepsis/septic shock: ENBS and optimal sample size of a trial, according to model for the synthesis of effectiveness evidence (see Table** 2 **for detailed specification of the models)**

**Scenarios for alternative synthesis models of effectiveness evidence**	**Maximum ENBS**	**Optimal sample size per arm (** n* **)**
**M1**	£ 136,703,882	1900
**M2**	£ 687,441,146	1200
**M3**	£ 1,010,953,361	800
**M4a**	£ 605,931,859	900
**M4b**	£ 365,050,246	800

The ENBS provides the societal payoff to the proposed research. It is computed as the difference between the value of sample information (EVSI) for a particular research design and the costs of sampling. IVIG, intravenous immunoglobulin; ENBS: expected net benefit of sampling.

## Discussion

Despite the existence of a large number of RCTs and numerous previous systematic reviews and meta-analyses of the relative effectiveness of IVIG, there remains controversy surrounding the value of IVIG as an adjunctive treatment in severe sepsis. Our study is the first to combine a formal systematic review of clinical effectiveness together with other epidemiological, resource use and quality of life data in order to robustly assess both the cost-effectiveness of IVIG based on existing evidence as well as the value of conducting further research.

Within this work, we re-analysed existing relative effectiveness RCT evidence and conducted a new meta-analysis, the first to simultaneously allow for type of IVIG (IVIG or IVIGAM), choice of control (no treatment or albumin), study quality/publication bias and other potential covariates. Our results indicated that treatment with IVIG may be associated with lower mortality but the evidence base shows a large degree of heterogeneity between individual studies. Given it was unclear what was the relevant source of heterogeneity in the evidence base, alternative clinical effectiveness models were evaluated.

Our results on the cost-effectiveness of IVIG appear within the borderline region of estimates considered to represent value for money in the NHS, but these results are associated with significant decision uncertainty and appear highly sensitive to the alternative clinical effectiveness models applied. Using an expected value of information framework, we established the value of collecting further information. Results show that a study collecting data on the relative effectiveness of IVIG (in comparison with standard care) appeared the most efficient research design to invest in. However, results on the value of conducting such research were also sensitive to the clinical effectiveness model used. Given that it was unclear what the clinical rationale for the effects explored was within each of the clinical effectiveness models and that the need for a further RCT exists, designing this study will be complex when uncertainties exist at this level.

### Policy implications

Our study did not find evidence that current guidance on the use of IVIG should change (that is IVIG should not be recommended for use in severe sepsis, unless further evidence becomes available to support its use). Although the EVI analyses suggested substantial potential value from a large, multicentre RCT evaluating the clinical effectiveness of IVIG in this population, there remain significant uncertainties around the design of a study (with respect to, for example, the dose or duration of therapy with IVIG). Without greater understanding of the existing variation (for example through a smaller-scale dosing RCT), there is a danger of investing in a large trial that is suboptimal (for example including a number of different dosing regimens), which may be a less efficient use of resources than deferring a definitive trial until we better understand the existing variation. Thus, our current recommendations are for research that focuses on filling the knowledge gaps that exist with a view to informing the design of a future multicentre RCT. These recommendations include: (i) research on the mechanism(s) of action of IVIG in severe sepsis (and on understanding the heterogeneity of the severe sepsis syndrome) - commencing with a rigorous review of existing research prior to embarking on any new studies and; (ii) dose-ranging/finding studies to identify dose, timing of dose and safety data to inform the intervention(s).

In addition to clarifying the results on clinical effectiveness, it may be that research informing other parameters is also worthwhile, especially with respect to data on the longer-term survival and costs of severe sepsis. This research could be conducted using relatively cheaper, non-RCT designs, for example using record linkage between existing databases or conducting a prospective cohort study providing that the period for which patients are observed is sufficiently long enough to capture the impact on costs for several years after the initial episode.

## Conclusions

Our study examined the evidence for the clinical effectiveness of IVIG in severe sepsis (including septic shock) and found significant heterogeneity (variation) between studies. An in-depth look at the potential sources for the heterogeneity identified publication bias and dosing regimen as possible explanatory factors. The rationale for the latter being a relevant factor was unclear, and for this reason we examined the implications of the existing heterogeneity and concluded it affected both the potential clinical and cost-effectiveness of this treatment and, hence, current recommendations for its use.

We assessed the value of a new, large, multicentre RCT to evaluate the clinical and cost-effectiveness of IVIG in this population and, while our analyses suggest that such research may be of value, there remains significant uncertainties around the design for such a study (with respect to, for example, the dose or duration of IVIG). We concluded that, prior to investing in a new RCT, further research is needed both into the mechanism(s) of action of IVIG and from a dose-ranging/finding study.

Our work illustrates the use of an explicit framework to quantify the value of investing in new research. This work also highlights that explicit consideration of the sources of heterogeneity in the current evidence base is key to informing the design of new research.

## Key messages

- The effect of IVIG on mortality is associated with a large degree of heterogeneity between individual studies. It was unclear what the relevant source of heterogeneity in the evidence base was.
- The cost-effectiveness of IVIG appears within the borderline region of estimates considered to represent value for money in the NHS, but these results are associated with significant uncertainty and appear highly sensitive to the alternative clinical effectiveness models applied.
- Collecting data on the relative effectiveness of IVIG appears an efficient research design in which to invest. However, the unclear clinical rationale for the heterogeneity in the evidence base suggests that, despite the need for a further RCT, designing this study will be complex when uncertainties exist at this level.

## Abbreviations

95%CrI: 95% credible intervals
AIC: Akaike information criterion
APACHE II: Acute Physiology and Chronic Health Evaluation II
CMPD: Case Mix Programme Database
DIC: deviance information criteria
Dres: residual deviance
ENBS: expected net benefit of sampling
EVI: expected value of information
EVPI: expected value of perfect information
EVPPI: partial EVPI
EVSI: expected value of sample information
HRQoL: health-related quality of life
HTA: Health Technology Assessment
ICER: incremental cost effectiveness ratio
ICNARC: Intensive Care National Audit & Research Centre
IVIG: intravenous immunoglobulin
IVIGAM: immunoglobulin M-enriched polyclonal IVIG
LOS: length of stay
NHS: National Health Service
OR: odds ratio
PSS: Personal Social Services
QALY: quality-adjusted life years
RCT: randomised controlled trial
SC: standard care
SD: standard deviation
SE: standard error
SICS: Scottish Intensive Care Society
UK: United Kingdom
